# Author Correction: Molecular mechanism for the control of virulent *Toxoplasma gondii* infections in wild-derived mice

**DOI:** 10.1038/s41467-019-09648-2

**Published:** 2019-04-04

**Authors:** Mateo Murillo-León, Urs B. Müller, Ines Zimmermann, Shishir Singh, Pia Widdershoven, Claudia Campos, Catalina Alvarez, Stephanie Könen-Waisman, Nahleen Lukes, Zsolt Ruzsics, Jonathan C. Howard, Martin Schwemmle, Tobias Steinfeldt

**Affiliations:** 1 Institute of Virology, Medical Center University of Freiburg, 79104 Freiburg, Germany; 2 Faculty of Medicine, University of Freiburg, 79104 Freiburg, Germany; 3 Faculty of Biology, University of Freiburg, 79104 Freiburg, Germany; 4 Institute for Genetics, University of Cologne, 50674 Cologne, Germany; 5 Department of Biology, University of Cologne, 50674 Cologne, Germany; 6 Fundação Calouste Gulbenkian, Instituto Gulbenkian de Ciencia, 2780-156 Oeiras, Portugal; 7 Department for Dermatology and Venereology, University Hospital of Cologne, 50937 Cologne, Germany; 8 Institute of Immunology, University Hospital Aachen, 52074 Aachen, Germany

Correction to: *Nature Communications* 10.1038/s41467-019-09200-2, published online 15 March 2019.

The original version of this Article contained an error in the Acknowledgements, which incorrectly omitted the following: ‘C.C., C.A., and J.C.H. were supported by the Fundação Calouste Gulbenkian through a grant from the Instituto Gulbenkian de Ciência and by the research infrastructure Congento, project LISBOA-01-0145-FEDER-022170, co-financed by Lisboa Regional Operational Programme (Lisboa 2020), under the Portugal 2020 Partnership Agreement, through the European Regional Development Fund (ERDF), and Foundation for Science and Technology (Portugal).’ This has been corrected in both the PDF and HTML versions of the Article.

