# Supplementary material for: Molecular mechanism for the control of virulent Toxoplasma gondii infections in wild-derived mice
Source: Nat Commun. 2019 Mar 15;10:1233. doi: 10.1038/s41467-019-09200-2 (PMC6420625; doi:10.1038/s41467-019-09200-2)
Supplement: Supplementary file 1 — Supplementary Information [file 41467_2019_9200_MOESM1_ESM.pdf]

Molecular mechanism for the control of virulent *Toxoplasma gondii* infections in wild-derived mice

Murillo León et al.

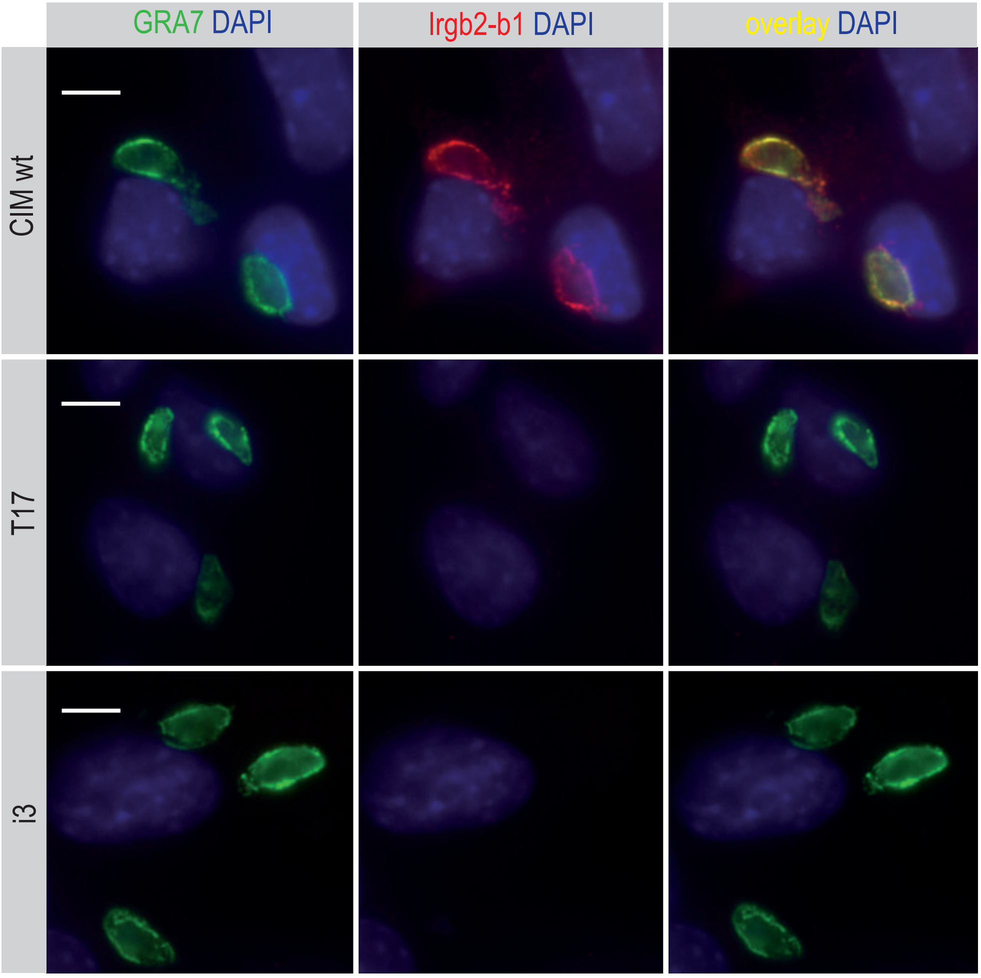

Supplementary Figure 1. Representative fluorescent images of *T. gondii* RHΔ*hxgprt*-derived vacuoles. CIM wt and Irgb2-b1$_{CIM}$ ko cells T17 and i3 have been stimulated with 200 U ml^{-1} IFNγ for 24 h and infected with RHΔ*hxgprt* at an MOI of 5. After 1.5 h, cells were prepared for immunofluorescence analysis as described in Methods. GRA7 in green (left hand panels), Irgb2-b1$_{CIM}$ in red (middle panels), and overlay (right hand panels) are shown. Nuclei (blue, all panels) stained with 4′,6-diamidino-2-phenylindole (DAPI, blue). All pictures for Irgb2-b1$_{CIM}$ were taken with the same exposure time. Scale bars, 5 µm.!

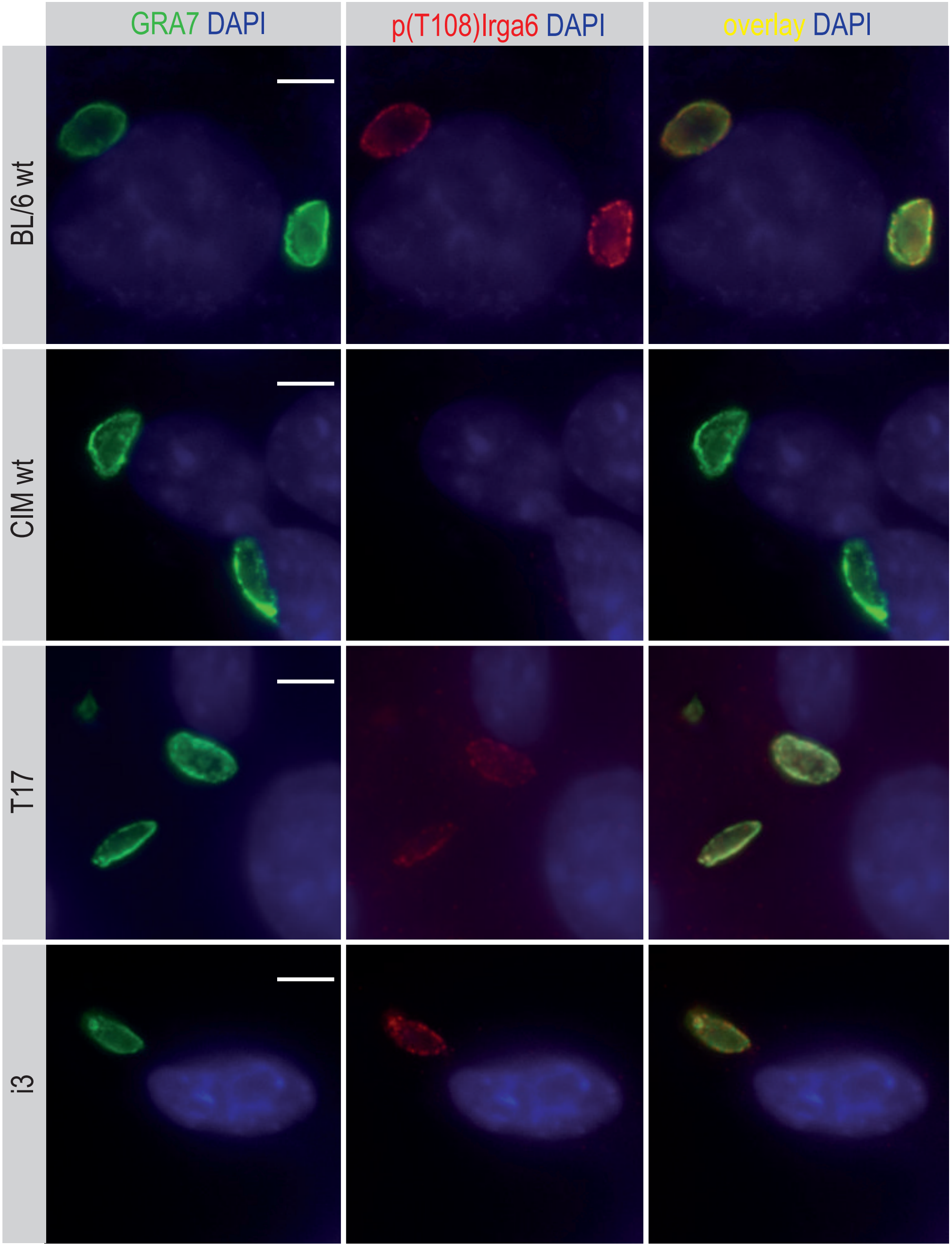

Supplementary Figure 2. Representative fluorescent images of *T. gondii* RHΔ*hxgprt*-derived vacuoles. CIM wt, BL/6 wt and Irgb2-b1$_{CIM}$ ko cells T17 and i3 have been stimulated with 200 U ml^{-1} IFNγ for 24 h and infected with RHΔ*hxgprt* at an MOI of 5. After 1.5 h, cells were prepared for immunofluorescence analysis as described in Methods. GRA7 (left hand panels, green), p(T108)Irga6 (middle panels, red), nuclei stained with 4´,6-diamidino-2-phenylindole (DAPI, blue). All pictures for p(T108)Irga6 were taken at the same exposure time. Scale bars, 5 µm.!

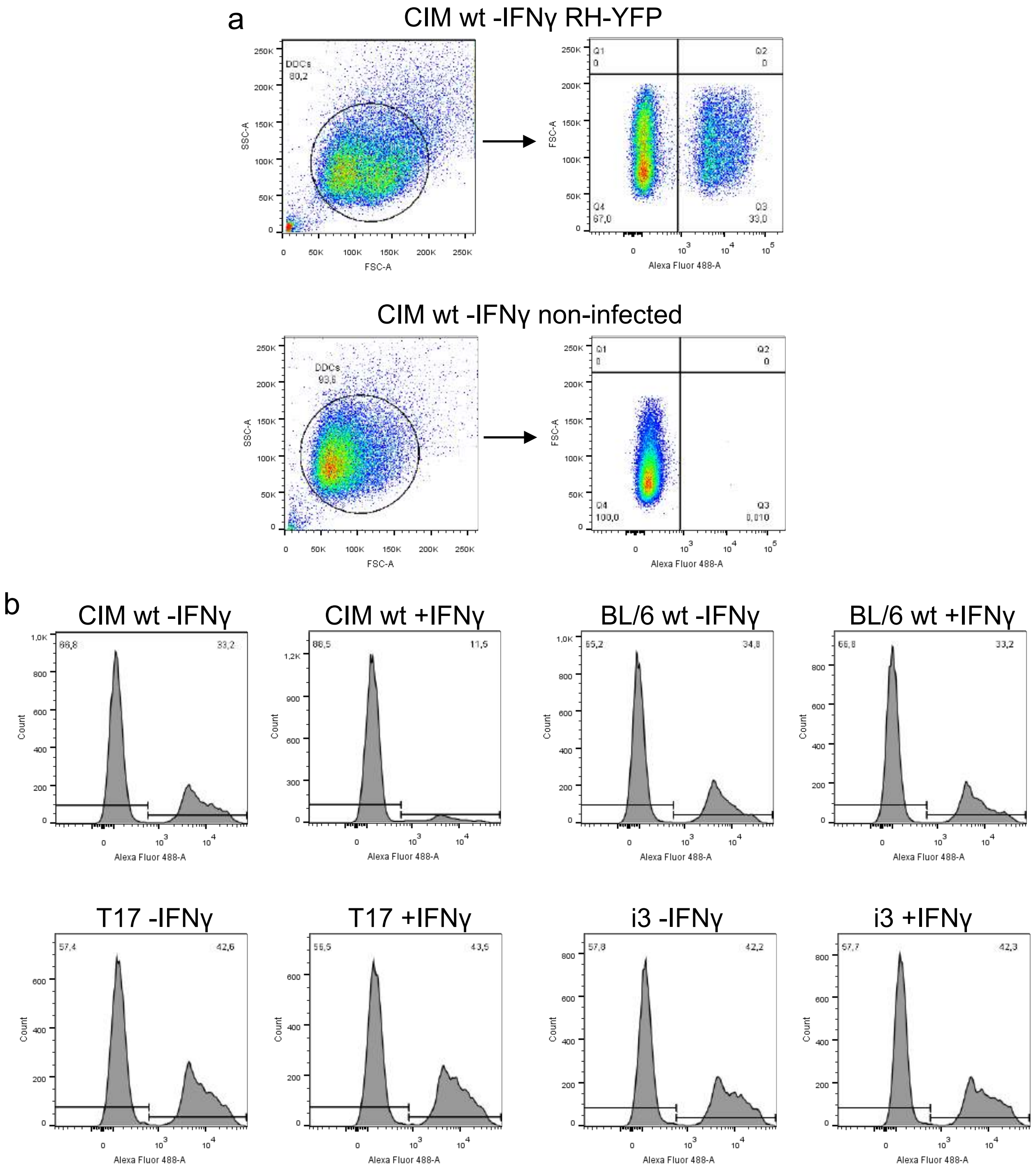

Supplementary Figure 3. Flow cytometric analysis of *T. gondii* replication. (a) *T. gondii* RH-YFP-infected (upper left hand panel) and non-infected (lower left hand panel) CIM wt cells were gated based on SSC-A vs FSC-A plots. Within these gates, the *T. gondii*-positive cells (Alexa Fluor 488-A) were determined and depicted in Q3 in a quadrant plot (right hand panels). (b) Cell lines were either stimulated with 100 U ml^{-1} IFNγ for 24 h or left unstimulated and subsequently infected with *T. gondii* RH-YFP at an MOI of 3. 24 h post infection, cells were prepared for FACS analysis and YFP-expression (Alexa Fluor 488-A) by *T. gondii* tachyzoites was used to calculate the percentage of inhibition of replication in presence of IFNγ as described in Methods.

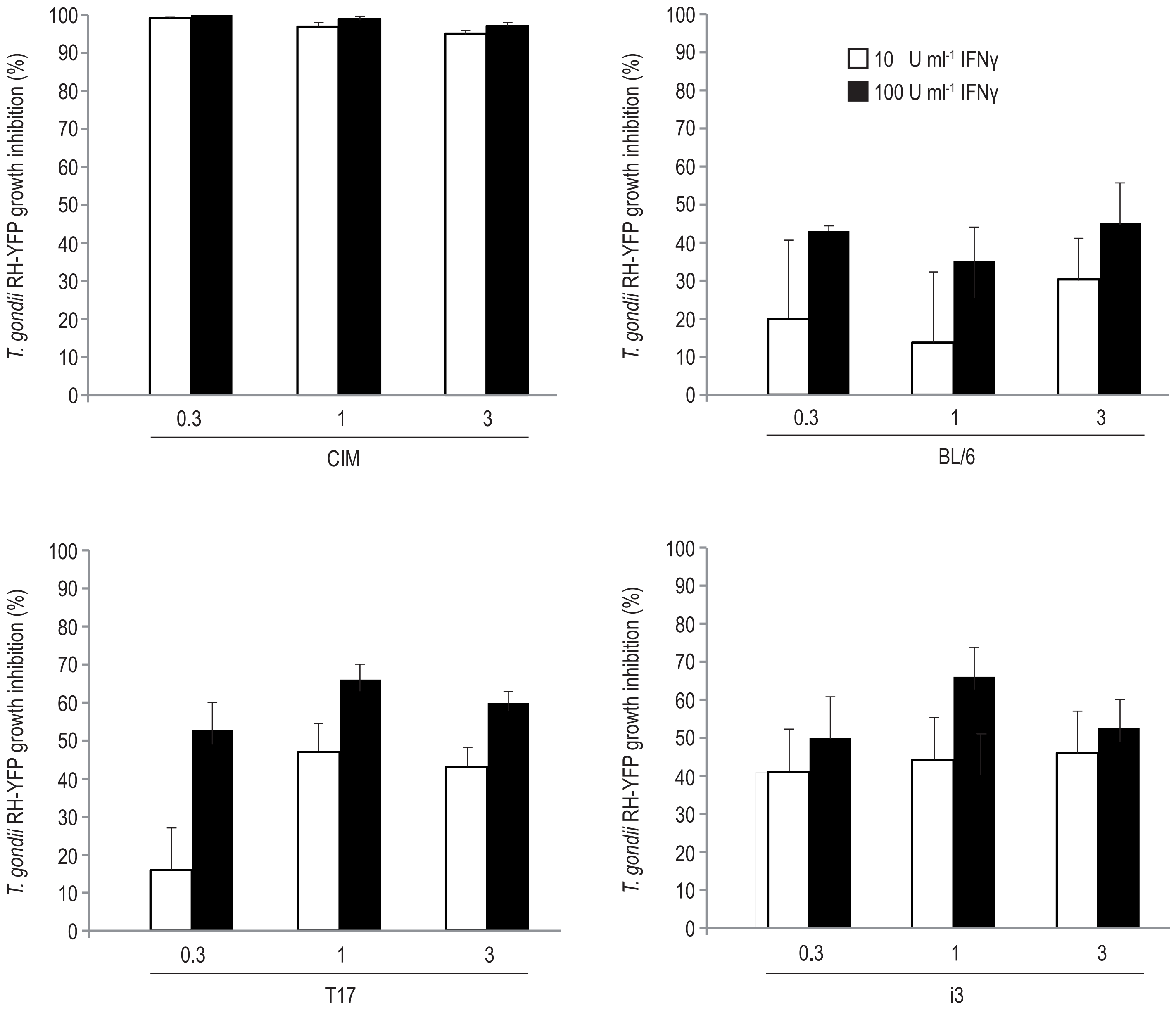

Supplementary Figure 4. Inhibition of *T. gondii* growth in CIM DDCs by IFNγ. CIM DDCs were induced with 10 or 100 U ml^{-1} IFNγ for 24 h and infected with RH-YFP at an MOI of 0.3, 1 or 3. After 24 h, ^{3}H-uracil was added and growth inhibition calculated 24 h later relative to cells not induced with IFNγ. Growth restriction of *T. gondii* RH-YFP in CIM wt cells is lost in *Irgb2-b1*$_{CIM}$ ko cells. Error bars represent standard deviations of triplicate values.

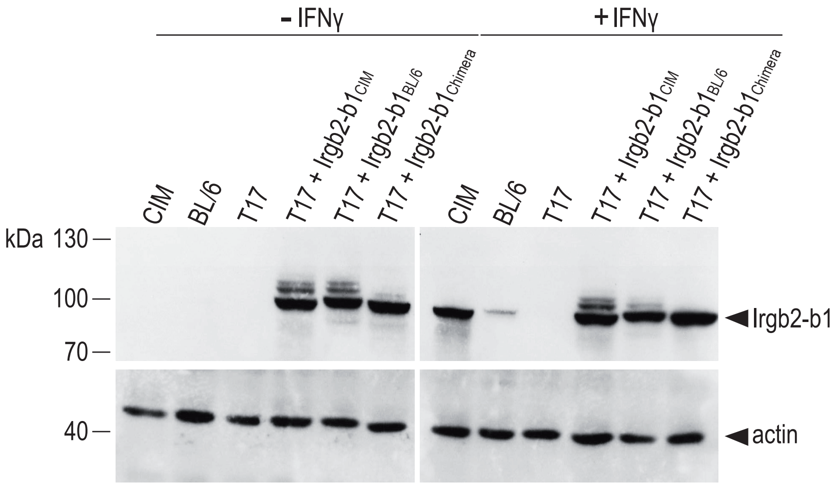

Supplementary Figure 5. Expresion levels of Irgb2-b1 variants in complemented T17 cells. Western blot of detergent lysates from CIM wt, BL/6 wt and *Irgb2-b1*$_{CIM}$ ko DDCs (T17) complemented with *Irgb2-b1* variants stimulated or not for 24 h with 200 U ml^{-1} IFNγ. The signal representing Irgb2-b1 in complemented cells is visible in stimulated and unstimulated cells whereas Irgb2-b1$_{CIM}$ and Irgb2-b1$_{BL/6}$ in wt cells is only detectable upon IFNγ stimulation. In no case Irgb2-b1$_{CIM}$ is detectable in *Irgb2-b1*$_{CIM}$ ko cells (upper panel). Actin serves as loading control (lower panel). All tracks were run on a single gel; the vertical white line indicates division of the gel for immunostaining with two different antibodies.

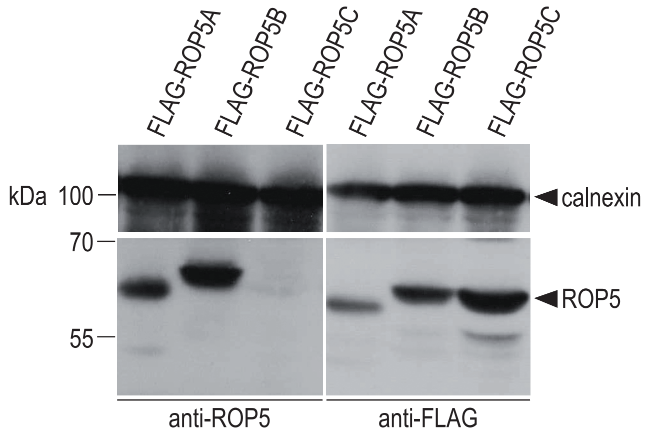

Supplementary Figure 6. The anti-ROP5 antibody 3E2 is isoform-specific. Western blot of detergent lysates from HEK293T cells transfected with FLAG-tagged ROP5 isoforms A, B or C. The ROP5 antibody 3E2 clearly detects ROP5A and B but not ROP5C (lower left hand panel). The anti-FLAG antibody indicates expression of all three ROP5 isoforms in the same cell lysates (lower right hand panel). Calnexin serves as loading control (upper panels). All tracks were run on a single gel; the vertical white line indicates division of the gel for immunostaining with two different antibodies.

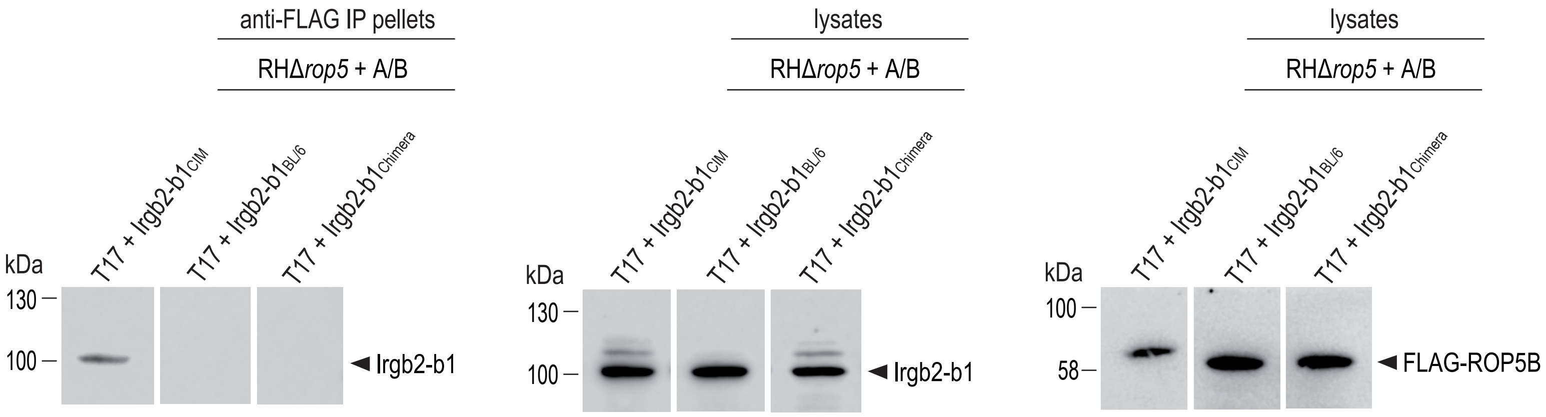

Supplementary Figure 7. Virulent ROP5B binds to Irgb2-b1$_{\mathbf{CIM}}$ but not Irgb2-b1$_{\mathbf{BL/6}}$ or Irgb2-b1$_{\mathbf{Chimera}}$ in infected cells. T17 + Irgb2-b1$_{\mathrm{CIM}}$, T17 + Irgb2-b1$_{\mathrm{BL/6}}$ and T17 + Irgb2-b1$_{\mathrm{Chimera}}$ DDCs were infected with *T. gondii* transgenic strain RHΔ*rop5* + A/B, expressing HA-tagged ROPA and FLAG-tagged ROP5B. Only Irgb2-b1$_{\mathrm{CIM}}$ but not Irgb2-b1$_{\mathrm{BL/6}}$ or Irgb2-b1$_{\mathrm{Chimera}}$ could be co-immunoprecipitated using a FLAG-specific antibody (left hand panel). The middle and right hand panels display amounts of Irgb2-b1 and FLAG-ROP5B in the lysates used for immunoprecipitation. All tracks were run on a single gel; vertical white lines indicate excision of irrelevant tracks.

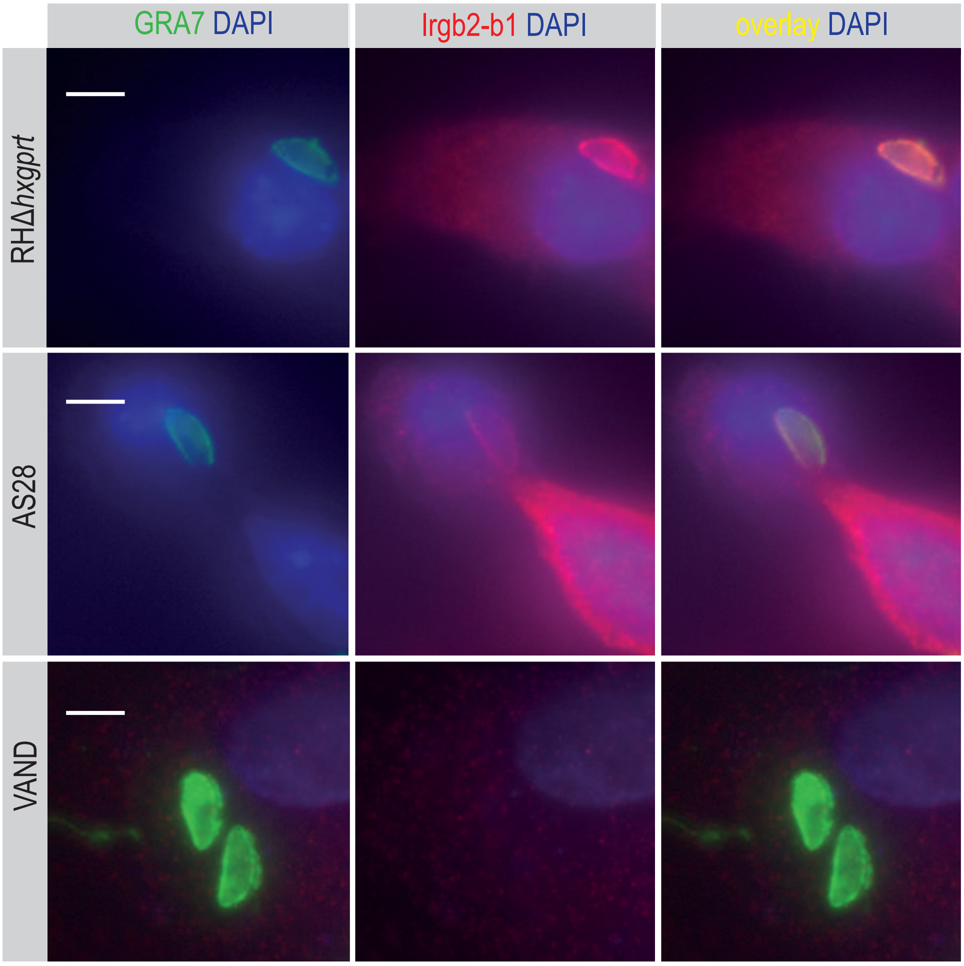

Supplementary Figure 8. Representative fluorescent images of *T. gondii* RHΔ*hxgprt*-, AS28- and VAND-derived vacuoles. CIM wt cells have been stimulated with 200 U ml^{-1} IFNγ for 24 h and infected with RHΔ*hxgprt*, AS28 or VAND at an MOI of 5. After 3 h, cells were prepared for immunofluorescence analysis like described in Methods. GRA7 (left hand panels, green), Irgb2-b1$_{CIM}$ (middle panels, red), nuclei stained with 4′,6-diamidino-2-phenylindole (DAPI, blue). All pictures for Irgb2-b1$_{CIM}$ were taken at the same exposure time. Scale bars, 5 µm.

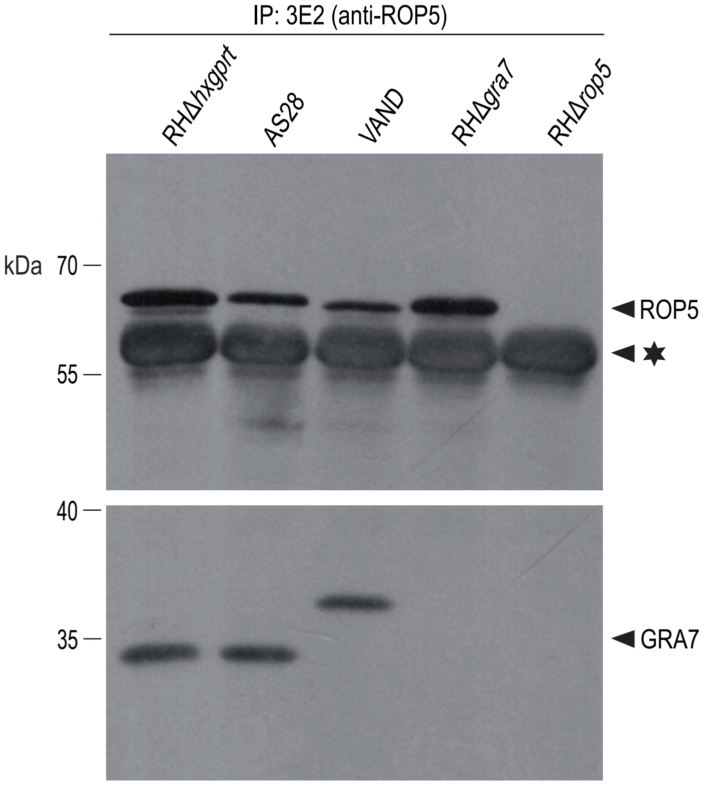

Supplementary Figure 9. ROP5 and GRA7 levels in tachyzoite detergent lysates. Western blot after immunoprecipitation of ROP5 using a ROP5-specific antibody (3E2) from detergent lysates of indicated *T. gondii* tachyzoites. ROP5 (upper panel) levels are comparable between *T. gondii* strains, RHΔ*rop5* served as control to confirm protein identity. GRA7 co-immunoprecipitated with ROP5 in case of RHΔ*hxgprt* and VAND but missing with RHΔ*rop5*. In case of AS28, GRA7 association with ROP5 has yet to be determined (lower panel). RHΔ*gra7* served as control to confirm protein identity. The star indicates heavy chain of the antibody used for immunoprecipitation.

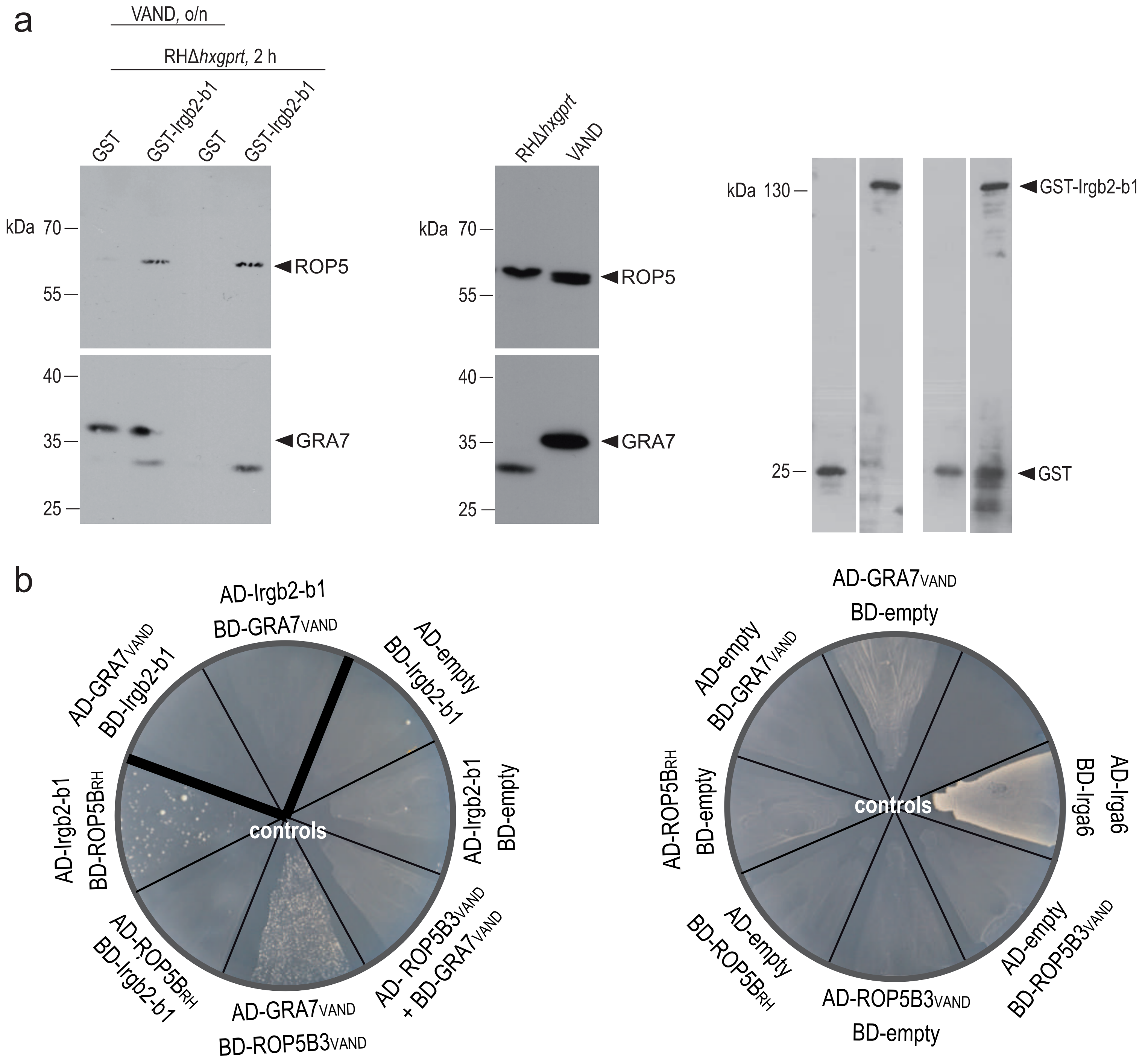

Supplementary Figure 10. VAND GRA7 is not associated with Irgb2-b1$_{CIM}$. (a) Pull-down of ROP5 and GRA7 by GST-Irgb2-b1$_{CIM}$ or GST alone. Pre-incubation with VAND detergent lysates (upper left hand panel, lanes 1 and 2) has no influence on RHΔ*hxgprt*-derived ROP5 association with GST-Irgb2-b1$_{CIM}$ (upper left hand panel, lanes 2 and 4). RHΔ*hxgprt*-derived GRA7 association with GST-Irgb2-b1$_{CIM}$ is dependent on ROP5 (left hand panels, lanes 2 and 4), VAND-derived GRA7 is associated with GST (lower left hand panel, lanes 1 and 2). The middle panels show ROP5 (upper panel) and GRA7 (lower panel) levels in tachyzoite lysates. The right hand panels indicate input of GST and GST-Irgb2-b1$_{CIM}$ proteins in the pull-down. All tracks were run on a single gel; vertical white lines indicate excision of irrelevant tracks. (b) VAND GRA7 does not directly interact with Irgb2-b1$_{CIM}$ in a Yeast Two-Hybrid approach. Proteins were expressed either as fusion to a transcriptional activation domain (AD) from pGAD-C3 or to a DNA-binding domain (BD) from pGBD-C3. Colony growth under 3DO conditions is indicative of protein:protein-interaction. Bold black lines separate samples from negative controls.

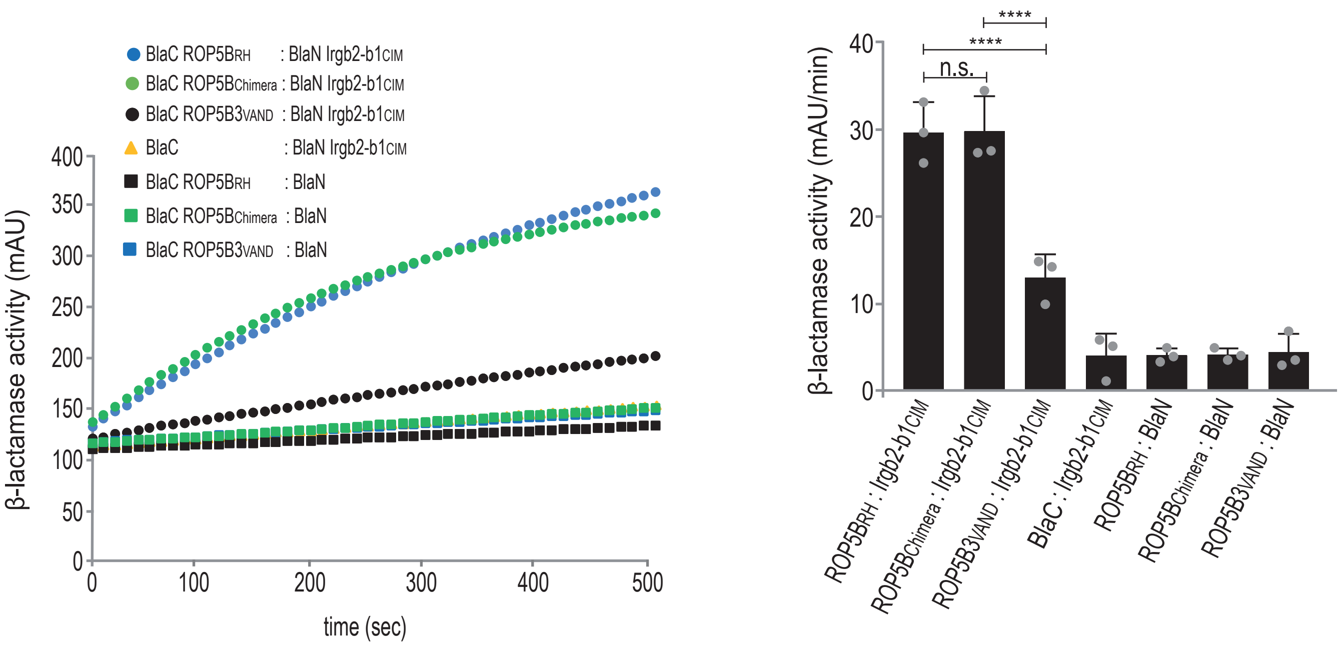

Supplementary Figure 11. ROP5B uses a highly polymorphic surface to bind to Irgb2-b1$_{\mathbf{CIM}}$. Protein-fragment Complementation Assay. Proteins were fused to N-terminal (BlaN) or C-terminal (BlaC) fragments of the reporter protein *TEM-1* β-lactamase. Error bars indicate the mean and SEM of three independent experiments (right hand panel). One-way ANOVA followed by Tukey´s multiple comparison was used to test differences between groups. ****p < 0.0001; n.s., not significant. The kinetic of the β-lactamase reaction is shown for one representative experiment (left hand panel). In comparison to ROP5B$_{\mathrm{RH}}$ and ROP5B$_{\mathrm{Chimera}}$, binding of ROP5B3$_{\mathrm{VAND}}$ to Irgb2-b1$_{\mathrm{CIM}}$ is significantly reduced.

Supplementary Table 1

Primers	Sequence	Construct
CIM BL6 Chimera internal fw	5´-GAACTTGCCAAAGCTATTGTGCAAATG	CIM-BL6$_{Chimera}$ fragment
CIM BL6 Chimera internal bw	5´-CATTTGCACAATAGCTTTGGCAAGTTC	CIM-BL6$_{Chimera}$ fragment
Irgb2-b1 CIM fw	5´-CCCCCCGGATCCATGGATCAGACTTCCTCTTCTACATCCC	pGEX-4T-2-Irgb2-b1$_{CIM}$
Irgb2-b1 CIM bw	5´-CCCCCCGTCGACTCACAGCTCCATTCCTGTTTCC	pGEX-4T-2-Irgb2-b1$_{CIM}$
Irgb2-b1 BL6 fw	5´-CCCCCGTCGACATGGATCAGACTTCCTCTTCTACATCCC	pGEX-4T-2-Irgb2-b1 $_{BL6}$
Irgb2-b1 BL6 bw	5´-GCGGCCGCTCACAGCTCCATTCCTGTTTCCCA	pGEX-4T-2-Irgb2-b1$_{BL/6}$
Irgb2 CIM bw	5´-CCCCCCGTCGACTCACTGCTCTGTGAAAAGCTCTTCTTTACTG	pGEX-4T-2-Irgb2$_{CIM}$
Irgb1 CIM fw	5´-CCCCCCGGATCCATGCATCCTCCATTGAACACAGCTACATGC	pGEX-4T-2-Irgb1 $_{CIM}$
Irgb2-b1 CIM fw YTH	5´-CCCCCCCATATGGATCAGACTTCCTCTTCTACATC	pGAD-C3-Irgb2-b1$_{CIM}$ and pGBD-C3-Irgb2-b1$_{CIM}$
Irgb2-b1 CIM bw YTH	5´-CCCCCCCTCGAGTCACAGCTCCATTCCTGT	pGAD-C3-Irgb2-b1$_{CIM}$ and pGBD-C3-Irgb2-b1$_{CIM}$
Irgb2-b1 BL6 fw YTH	5´-CCCCCCCATATGGGTCAGACTTCCTCTTCTAACA	pGAD-C3-Irgb2-b1$_{BL/6}$ and pGBD-C3-Irgb2-b1$_{BL/6}$
Irgb2-b1 CIM fw PCA	5´-CCCCCCGCTAGCATGGATCAGACTTCCTCTTCTACATCCCCT	BlaN-Irgb2-b1$_{CIM}$ and Blac-C3-Irgb2-b1$_{CIM}$
Irgb2-b1 CIM bw PCA	5´-CCCCCCGCGGCCGCTCACAGCTCCATTCCTGTTTCCCA	BlaN-Irgb2-b1$_{CIM}$ and BlaC-Irgb2-b1$_{CIM}$
Irgb2-b1 BL6 fw PCA	5´-CCCCCCGCTAGCATGGGTCAGACTTCCTCTTCTACA	BlaN-Irgb2-b1$_{BL/6}$ and BlaC-Irgb2-b1$_{BL/6}$
BL6 b2-b1 BL6 fw PCA	5´-CCCCCCGCTAGCATGGGTCAGACTTCCTCTTCTACA	BlaN-Irgb2-b1$_{BL/6}$ and BlaC-Irgb2-b1$_{BL/6}$
Irgb2-b1 CIM BL6 Chimera fw PCA	5'-CCCCCCGCTAGCATGGATCAGACTTCCTCTTCTACATC	BlaN-Irgb2-b1$_{Chimera}$ and BlaC-Irgb2-b1$_{Chimera}$
Irgb2-b1 CIM BL6 Chimera bw PCA	5'-CCCCCCGCGGCCGCTCACAGCTCCATTCCTGTTTCC	BlaN-Irgb2-b1$_{Chimera}$ and BlaC-Irgb2-b1$_{Chimera}$
RH ROP5A fw PCA	5'-CCCCCCGCTAGCATGGCGACGAAGCTCGCTAG	BlaN-ROP5A $_{RH}$ and BlaC-ROP5A $_{RH}$
RH ROP5A bw PCA	5'-CCCCCCGCGGCCGCTCAAGCGACTGAGGGCGC	BlaN-ROP5A $_{RH}$ and BlaC-ROP5A $_{RH}$
RH ROP5B fw PCA	5´-CCCCCCGCTAGCATGGCGACGAAGCTTGCTAGACTAGC	BlaN-ROP5B $_{RH}$ and BlaC-ROP5B $_{RH}$
RH ROP5B bw PCA	5´-CCCCCCGCGGCCGCTCAAGCGACTGAGGGCGCAGC	BlaN-ROP5B $_{RH}$ and BlaC-ROP5B $_{RH}$
RH ROP5C fw PCA	5´-CCCCCCGCTAGCATGGCGACGAAGCTCGCTAGACTAG	BlaN-ROP5C $_{RH}$ and BlaC-ROP5C $_{RH}$
RH ROP5C bw PCA	5´-CCCCCCGCGGCCGCTCAAAGCGACTGAGGGCGCAGCAA	BlaN-ROP5C $_{RH}$ and BlaC-ROP5C $_{RH}$
VAND ROP5B3 fw PCA	5´-CCCCCCGCTAGCATGGCGACGAAGCTCGCTAGA	BlaN-ROP5B3 $_{VAND}$ and BlaC-ROP5B3$_{VAND}$
VAND ROP5B3 bw PCA	5´-CCCCCCGCGGCCGCTCAAGCGACTGAGGGCGCAG	BlaN-ROP5B3$_{VAND}$ and BlaC-ROP5B3$_{VAND}$
VAND ROP5B3 fw PCA	5´-CCCCCCGCTAGCATGGCGACGAAGCTCGCTAGA	BlaN-ROP5B3$_{Chimera}$ and BlaC-ROP5B3$_{Chimera}$
VAND ROP5B3 bw PCA	5´-CCCCCCGCGGCCGCTCAAGCGACTGAGGGCGCAG	BlaN-ROP5B3$_{Chimera}$ and BlaC-ROP5B3$_{Chimera}$
VAND ROP5A fw YTH	5'-CCCCCCCATATGGCGACGAAGCTCGCTAG	pGAD-C3-ROP5A$_{VAND}$ and pGBD-C3-ROP5A$_{VAND}$
VAND ROP5A bw YTH	5'-CCCCCCCTCGAGTCAAGCGACTGAGGGCGC	pGAD-C3-ROP5A$_{VAND}$ and pGBD-C3-ROP5A$_{VAND}$
VAND ROP5B1 fw YTH	5'-CCCCCCCATATGGCGACGAAGCTCGCT	pGAD-C3-ROP5B1$_{VAND}$ and pGBD-C3-ROP5B1$_{VAND}$
VAND ROP5B1 bw YTH	5'-CCCCCCGGATCCTCAAGCGACTGAGGGCGC	pGAD-C3-ROP5B1$_{VAND}$ and pGBD-C3-ROP5B1$_{VAND}$
VAND ROP5B2 fw YTH	5'-CCCCCCCATATGGCGACGAAGCTCGCT	pGAD-C3-ROP5B2$_{VAND}$ and pGBD-C3-ROP5B2$_{VAND}$

VAND ROP5B2 bw YTH	5’-CCCCCCCTCGAGTCAAGCGACTGAGGGCGC	pGAD-C3-ROP5B2$_{VAND}$ and pGBD-C3-ROP5B2$_{VAND}$
VAND ROP5B3 fw YTH	5’-CCCCCCCATATGGCGACGAAGCTCGCTA	pGAD-C3-ROP5B3$_{VAND}$ and pGBD-C3-ROP5B3$_{VAND}$
VAND ROP5B3 bw YTH	5’-CCCCCCCTCGAGTCAAGCGACTGAGGGCGC	pGAD-C3-ROP5B3$_{VAND}$ and pGBD-C3-ROP5B3$_{VAND}$
VAND RH ROP5B3 Chimera fw YTH	5´-CCCCCCCATATGGCCACAAAGCTGGCCAG	pGAD-C3-ROP5B3$_{Chimera}$ and pGBD-C3-ROP5B3$_{Chimera}$
VAND RH ROP5B3 Chimera bw YTH	5´-CCCCCCGGATCCTCAAGCCACGCTAGGGGCG	pGAD-C3-ROP5B3$_{Chimera}$ and pGBD-C3-ROP5B3$_{Chimera}$
VAND GRA7 fw YTH	5’-CCCCCCCATATGGCCCGACACGCAATTTTTTTCGC	pGAD-C3-GRA7$_{VAND}$ and pGBD-C3-GRA7$_{VAND}$
VAND GRA7 bw YTH	5’-CCCCCCGGATCCCTACTGGCGGGCATCCTCCCC	pGAD-C3-GRA7$_{VAND}$ and pGBD-C3-GRA7$_{VAND}$
