# Supplementary material for: Molecular mechanism for the control of virulent Toxoplasma gondii infections in wild-derived mice
Source: Nat Commun. 2019 Mar 15;10:1233. doi: 10.1038/s41467-019-09200-2 (PMC6420625; doi:10.1038/s41467-019-09200-2)
Supplement: Supplementary file 3 — Reporting Summary [file 41467_2019_9200_MOESM3_ESM.pdf]

nature research

Corresponding author(s): PD Dr. Tobias Steinfeldt

Last updated by author(s): Feb 20, 2019

Reporting Summary

Nature Research wishes to improve the reproducibility of the work that we publish. This form provides structure for consistency and transparency in reporting. For further information on Nature Research policies, see Authors & Referees and the Editorial Policy Checklist.

Statistics

For all statistical analyses, confirm that the following items are present in the figure legend, table legend, main text, or Methods section.

n/a	Confirmed	
☐	☒	The exact sample size (*n*) for each experimental group/condition, given as a discrete number and unit of measurement
☐	☒	A statement on whether measurements were taken from distinct samples or whether the same sample was measured repeatedly
☐	☒	The statistical test(s) used AND whether they are one- or two-sided<br>*Only common tests should be described solely by name; describe more complex techniques in the Methods section.*
☒	☐	A description of all covariates tested
☐	☒	A description of any assumptions or corrections, such as tests of normality and adjustment for multiple comparisons
☐	☒	A full description of the statistical parameters including central tendency (e.g. means) or other basic estimates (e.g. regression coefficient) AND variation (e.g. standard deviation) or associated estimates of uncertainty (e.g. confidence intervals)
☐	☒	For null hypothesis testing, the test statistic (e.g. *F*, *t*, *r*) with confidence intervals, effect sizes, degrees of freedom and *P* value noted<br>*Give P values as exact values whenever suitable.*
☒	☐	For Bayesian analysis, information on the choice of priors and Markov chain Monte Carlo settings
☒	☐	For hierarchical and complex designs, identification of the appropriate level for tests and full reporting of outcomes
☒	☐	Estimates of effect sizes (e.g. Cohen's *d*, Pearson's *r*), indicating how they were calculated

Our web collection on statistics for biologists contains articles on many of the points above.

Software and code

Policy information about availability of computer code

Data collection	BD FACSDiva v6.1.3; Image Studio 3.1.4, LI-COR Biotechnology
Data analysis	Prism7, Graphpad Software, Inc.; AxioVision 4.8, Zeiss; FlowJo vX 10.0.7, Tree Star Inc.; MEGA (Molecular Evolutionary Genetics Analysis) 10.0

For manuscripts utilizing custom algorithms or software that are central to the research but not yet described in published literature, software must be made available to editors/reviewers. We strongly encourage code deposition in a community repository (e.g. GitHub). See the Nature Research guidelines for submitting code & software for further information.

Data

Policy information about availability of data

All manuscripts must include a data availability statement. This statement should provide the following information, where applicable:

- Accession codes, unique identifiers, or web links for publicly available datasets
- A list of figures that have associated raw data
- A description of any restrictions on data availability

The authors declare that all data supporting the findings of this study are available within the article and its Supplementary Information files, or are available from the authors upon request.

Field-specific reporting

Please select the one below that is the best fit for your research. If you are not sure, read the appropriate sections before making your selection.

☒ Life sciences ☐ Behavioural & social sciences ☐ Ecological, evolutionary & environmental sciences

For a reference copy of the document with all sections, see nature.com/documents/nr-reporting-summary-flat.pdf

Life sciences study design

All studies must disclose on these points even when the disclosure is negative.

Sample size

The sample sizes chosen for in vitro studies are based on experimental values obtained for similar experiments in recent and past publications. References are given in the Methods. In case of in vivo experiments, the pheotype (death of infected mice) was established in a preliminary experiment with a low number of animals (n=5). All infected animals succumbed to infection in a similar period of time compared to control animals . We chose much more mice fo the experiment (n=15 (control), n=25 and n=10 (control, n=19).

Data exclusions

No data were excluded from the analyses.

Replication

All attems at replication were successful.

Randomization

Animals included in the study were taken from random cages, as long as the age was appropriate to pursue the experimental procedures.

Blinding

Experiments including live animals did not include blinding. During i.p. infections, it was possible to know if the animal being infected was part of the control group or the experimental group.
Immunofluorescence analyses were done in a double-blind procedure.

Reporting for specific materials, systems and methods

We require information from authors about some types of materials, experimental systems and methods used in many studies. Here, indicate whether each material, system or method listed is relevant to your study. If you are not sure if a list item applies to your research, read the appropriate section before selecting a response.

Materials & experimental systems

n/a	Involved in the study	
☐	☒	Antibodies
☐	☒	Eukaryotic cell lines
☒	☐	Palaeontology
☐	☒	Animals and other organisms
☒	☐	Human research participants
☒	☐	Clinical data

Methods

n/a	Involved in the study	
☒	☐	ChIP-seq
☐	☒	Flow cytometry
☒	☐	MRI-based neuroimaging

Antibodies

Antibodies used

commercially available antibodies:

goat anti-GST, GE Healthcare 27457701, lot 325903
mouse anti-FLAG M2, Sigma F3165, lot SLBT6752
mouse anti-actin, Sigma A3853, lot 114M4816V
Alexa Fluor 488 donkey anti-rat, Thermo Fisher Scientific A21208, lot 1932496
Alexa Fluor 555 donkey anti-rabbit, Thermo Fisher Scientific A31572, lot 2017396
rabbit anti-mouse HRP, Jackson Immuno Research Laboratories 315-035-045, lot 113728
goat anti rabbit HRP, Jackson Immuno Research Laboratories 111-035-045, lot 139080
donkey anti-goat HRP, Jackson Immuno Research Laboratories 705-035-147, lot 128625
goat anti-rat HRP, Jackson Immuno Research Laboratories 112-035-003, lot ???

other antibodies:

3E2 mouse monoclonal antibody against ROP5: Fleckenstein et al., 2012, PlosBiology; Hermanns et al., 2016, Cellular Microbiology

affinity-purified rabbit sera 87558 against (pT108)Irga6: Steinfeldt et al., 2010, PlosBiology; Fleckenstein et al., 2012, PlosBiology; Hermanns et al., 2016, Cellular Microbiology

10D7 and 10E7 mouse monoclonal antibodies against Irga6: Papic et al., 2008, JBC; Khaminets et al., 2010, Cellular Microbiology;

Steinfeldt et al., 2010, PlosBiology; Fleckenstein et al., 2012, PlosBiology; Hermanns et al., 2016, Cellular Microbiology

B34 mouse monoclonal antibody against Irgb6: Carlow et al., 1998, Journal of Immunology; Khaminets et al., 2010, Cellular Microbiology; Steinfeldt et al., 2010, PlosBiology; Hermanns et al., 2016, Cellular Microbiology

940/6 rabbit antiserum against Irgb10: Hermanns et al., 2016, Cellular Microbiology

2078 rabbit antiserum against Irgd: Martens et al., 2004, Journal of Immunology; Khaminets et al., 2010, Cellular Microbiology; Hermanns et al., 2016, Cellular Microbiology

954/1-C15A rabbit antiserum against a conserved Irgb-tandem C-terminal peptide: Lilue et al., 2013, eLife

3.1.2 and 2.4.21 rat monoclonal antibodies against T. gondii GRA7: Hermanns et al., 2016, Cellular Microbiology

Validation

goat anti-GST: https://www.gelifesciences.com/en/ar/shop/chromatography/resins/affinity-tagged-protein/anti-gst-antibody-p-06000#overview

mouse anti-FLAG M2: https://www.sigmaaldrich.com/catalog/product/sigma/f3165?lang=de&region=DE

mouse anti-actin: https://www.sigmaaldrich.com/catalog/product/sigma/a3853?lang=de&region=DE

The following antibodies have been profiled using ko cells and ko T. gondii if available, or bacterially expressed and purified recombinant proteins:

3E2 mouse monoclonal antibody against ROP5: Fleckenstein et al., 2012, PlosBiology; Hermanns et al., 2016, Cellular Microbiology

affinity-purified rabbit sera 87558 against (pT108)Irga6: Steinfeldt et al., 2010, PlosBiology; Fleckenstein et al., 2012, PlosBiology; Hermanns et al., 2016, Cellular Microbiology

10D7 and 10E7 mouse monoclonal antibodies against Irga6: Papic et al., 2008, JBC; Khaminets et al., 2010, Cellular Microbiology; Steinfeldt et al., 2010, PlosBiology; Fleckenstein et al., 2012, PlosBiology; Hermanns et al., 2016, Cellular Microbiology

B34 mouse monoclonal antibody against Irgb6: Carlow et al., 1998, Journal of Immunology; Khaminets et al., 2010, Cellular Microbiology; Steinfeldt et al., 2010, PlosBiology; Hermanns et al., 2016, Cellular Microbiology

940/6 rabbit antiserum against Irgb10: Hermanns et al., 2016, Cellular Microbiology

2078 rabbit antiserum against Irgd: Martens et al., 2004, Journal of Immunology; Khaminets et al., 2010, Cellular Microbiology; Hermanns et al., 2016, Cellular Microbiology

954/1-C15A rabbit antiserum against a conserved Irgb-tandem C-terminal peptide: Lilue et al., 2013, eLife

3.1.2 and 2.4.21 rat monoclonal antibodies against T. gondii GRA7: Hermanns et al., 2016, Cellular Microbiology

Eukaryotic cell lines

Policy information about cell lines

Cell line source(s)

HEK293T cells (ATTC; CRL-3216), Human foreskin fibroblasts (HS27, ATCC; CRL-1634), diaphragm-derived cells (DDC) derived from C57BL/6 and CIM mice (Lilue et al. eLife 2013;2:e01298. DOI: 10.7554/eLife.01298)

Authentication

HEK293T cells and Human foreskin fibroblasts were authenticated morphologically by light microscopy. C57/BL/6 and CIM diaphram-derived cells (DDC) were authenticated by Western blot (CIM DDCs and BL/6 DDCs show different expression levels of a marker protein). Otherwise, none of the cell lines used were authenticated any further.

Mycoplasma contamination

All cell lines used in this study were tested negative for mycoplasma contamination.

Commonly misidentified lines
(See ICLAC register)

No misidentified cell lines were used in this study.

Animals and other organisms

Policy information about studies involving animals; ARRIVE guidelines recommended for reporting animal research

Laboratory animals

Mus musculus domesticus C57BL/6 and Mus musculus castaneus CIM mice with ages ranging from 2-4 months were included in the study. Females and males were used indiscriminately.

Wild animals

not applicable

Field-collected samples	not applicable
Ethics oversight	Cologne (Germany): Bezirksregierung Köln, Germany, LANUV Nordrhein-Westfalen Permit No. 44.07.189<br>Oeiras (Portugal): Portaria 1005/92

Note that full information on the approval of the study protocol must also be provided in the manuscript.

Flow Cytometry

Plots

Confirm that:

☒ The axis labels state the marker and fluorochrome used (e.g. CD4-FITC).

☒ The axis scales are clearly visible. Include numbers along axes only for bottom left plot of group (a 'group' is an analysis of identical markers).

☒ All plots are contour plots with outliers or pseudocolor plots.

☒ A numerical value for number of cells or percentage (with statistics) is provided.

Methodology

Sample preparation	For FACS analysis, diaphragm-derived cells (DDC) were seeded in 12 well-plates. 24 h post infection cells were trypsinized, washed 2x with PBS containing 3 % FCS (PBS/FCS) and resuspended in 400 µl PBS/FCS containing 1 % PFA. After 15 min incubation at RT, fixed cells were washed 2x with PBS/FCS, resuspended in 400 µl PBS/FCS and analyzed.
Instrument	FACSCanto II flow cytometer (BD Biosciences)
Software	BD FACSDiva v6.1.3; FlowJo vX 10.0.7, Tree Star Inc.
Cell population abundance	not applicable
Gating strategy	The gating strategy is provided in Supplementary Figure 3.

☒ Tick this box to confirm that a figure exemplifying the gating strategy is provided in the Supplementary Information.
